# Interleukins in the Pathogenesis of Warts: Insight from the Last Decade—A Narrative Review

**DOI:** 10.3390/jcm14062057

**Published:** 2025-03-18

**Authors:** Clara Matei, Laura Sorina Diaconu, Mircea Tampa

**Affiliations:** 1 Department of Dermatology, “Carol Davila” University of Medicine and Pharmacy, 020021 Bucharest, Romania; 2 Department of Internal Medicine III and Gastroenterology, “Carol Davila” University of Medicine and Pharmacy, 020021 Bucharest, Romania; 3 Department of Internal Medicine III and Gastroenterology, Emergency University Hospital, 050098 Bucharest, Romania; 4 Department of Dermatology, “Victor Babes” Clinical Hospital for Infectious Diseases, 030303 Bucharest, Romania

**Keywords:** HPV, warts, interleukin, immune response

## Abstract

Human papillomavirus (HPV) is the etiological agent of a wide spectrum of diseases, from benign lesions to neoplasms. In most cases, in the first few years after infection, viral clearance occurs; however, in some cases, the infection remains persistent, allowing the progression of the lesions. The host immune response plays a key role in the resolution of the infection. The immune response to HPV is regulated by the dynamic interaction between numerous interleukins that exert pro- or anti-inflammatory effects. The role of interleukins in malignant lesions caused by HPV has been intensively studied, but in the case of benign lesions including warts, data are limited. This review compiles data from the last 10 years on the involvement of interleukins in the pathogenesis of warts, with the aim of providing new perspectives on this topic. Elucidating the role of interleukins will not only increase our knowledge of the pathogenesis of HPV infection but will also provide the foundation for the development of new therapies.

## 1. Introduction

**Figure 1:**
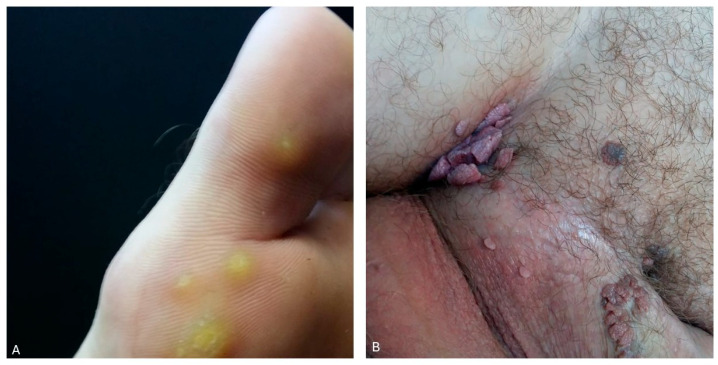
(**A**) Plantar warts. (**B**) Genital warts (personal collection).

Human papillomaviruses (HPVs) are non-enveloped DNA viruses classified into five genera (alpha, beta, gamma, mu, and nu) comprising over 200 types with a tropism for basal epidermal keratinocytes or squamous mucous membranes [1,2]. The beta, gamma, mu, and nu genera cause cutaneous infections, while the alpha genus leads to infections affecting both the skin and mucous membranes. HPVs cause a wide range of conditions, from benign to premalignant or malignant, due to the diversity of HPV types. Warts are the most common lesions caused by HPVs. On average, 10% of the population will develop warts at some point [3,4,5]. These benign lesions are classified based on their location and clinical aspects into several types, including common, plane, flat, palmar, plantar, and genital warts (condylomata acuminata) [6,7] (Figure 1). In most cases, diagnosis relies on the clinical appearance. However, in recent years, the role of dermoscopy in the diagnosis of cutaneous warts has gained attention. Dermoscopy reveals black, red, or brown globules and dots, which represent dilated capillaries surrounded by white halos [8].

At the same time, the role of HPV in carcinogenesis should be stressed, as it is the etiological agent for approximately 5% of cancers, according to the World Health Organization [4]. Over 90% of cervical cancers are caused by HPV infection [9]. E6 and E7 proteins are the main actors in the malignant transformation of cervical cells. Most newly acquired HPV infections and lesions are temporary and typically clear up within 1–2 years without treatment, thanks to a strong immune response. However, a small percentage of HPV infections persist beyond this period, increasing the risk of developing precancerous cervical lesions [10].

HPV is an intraepithelial virus that relies entirely on the complete differentiation process of keratinocytes for its replication [11,12,13]. Although the virus infects basal keratinocytes, the production of viral proteins and the expression of viral genes occur exclusively in the upper layers of the skin, specifically in the stratum spinosum and stratum granulosum, where keratinocytes are more differentiated [14]. Both innate immune response and acquired immunity play critical roles in defending against HPV. The Toll-like receptors (TLRs) 2, 3, 7, 8, and 9 recognize virus nucleic acids, leading to the release of a plethora of cytokines. An increased expression of these receptors is associated with viral clearance. Natural killer (NK) cells, but particularly CD4 and CD8 cells, are involved in the healing process of viral lesions [15,16]. Histopathological examinations have shown increased levels of CD4 and CD8 cells, as well as macrophages, in regressing skin lesions [17]. HPV remains confined to the epidermis or mucous membranes without reaching the bloodstream. The infectious process does not lead to cytolysis, and the epidermis and mucosal tissue act as barriers. All of these factors explain why HPV infection most often does not elicit a significant immune response [3]. It should be noted that HPV has the ability to evade immunity and to inhibit immune signaling pathways, which leads to viral persistence [14]. The ability of HPV to persist is linked to various mechanisms such as altering cytokine activity, suppressing interferon (IFN) pathways, disrupting antigen presentation, and decreasing the expression of adhesion molecules. These immune evasion strategies are primarily mediated by the E6 and E7 oncoproteins [18,19]. Both the Th1 responses that target intracellular pathogens and stimulate the inflammatory process and the Th2 responses that are directed toward extracellular pathogens and exhibit anti-inflammatory roles are involved in the pathogenesis of HPV infection. Cellular immunity is mandatory in the defense against HPV. CD4+ cells play a central role by releasing numerous cytokines/interleukins (ILs). There are two main subsets of CD4+ cells, Th1 and Th2, which induce a different pattern of cytokines. Th1 responses are primarily characterized by the production of IFN-γ, TNF-α, and IL-2, while Th2 responses involve the release of IL-4, IL-6, and IL-10. Today, it is known that Th1 responses correlate with HPV clearance [20]. The disruption of the immune response and its shift from a Th1 response to a Th2 response are associated with altered virus recognition by the immune system, facilitating the persistence of the virus for an indefinite period and resulting in the progression of the lesions [21].

IL-1α and β, IL-6, and IL-12 are involved in the adaptive immune response against HPV. Upon viral entry into the host, the virus is phagocytosed by dendritic cells and transported to lymph nodes, triggering the release of ILs with pro-inflammatory effects. According to a recent study, the IL-1α that is released by keratinocytes appears to play an important role in the resolution of HPV infection by mediating antigenic recognition [22].

**Figure 2:**
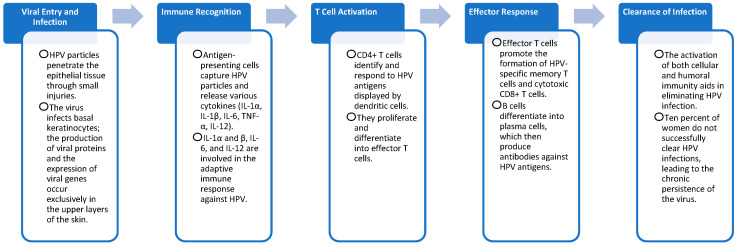
HPV infection steps [22].

The Th1 immune response is essential for an antiviral defense; therefore, ILs such as IL-2 activate immune cells, especially T cells. It seems that the shift from a Th1 to a Th2 response is identified in patients with persistent lesions or high-grade dysplastic lesions. and not in the case of primary infections. Increased levels of IL-6, IL-8, and TNF alter the antiviral response, allowing the virus to persist in infected cells [23]. Two other key interleukins involved in the pathogenesis of HPV infection are IL-12 and IL-23. IL-12 regulates the function of lymphocytes by inducing their differentiation into Th1 cells, while IL-23 is an activator of IL-17. IL-17 has a pro-inflammatory role and is involved in maintaining the epithelial barriers and in immunosurveillance (Figure 2) [24].

It should also be noted that Th2 cells secrete ILs (IL-4, IL-6, IL-8) that activate B cells and induce their transformation into plasma cells, which then produce antibodies against HPV antigens [22].

Deciphering the role of ILs in the occurrence of warts may provide new insights into HPV pathogenesis. In this review, we have collated the available data from the literature regarding ILs in patients with warts (cutaneous and anogenital warts) to offer a new perspective on this topic.

## 2. Materials and Methods

**Figure 3:**
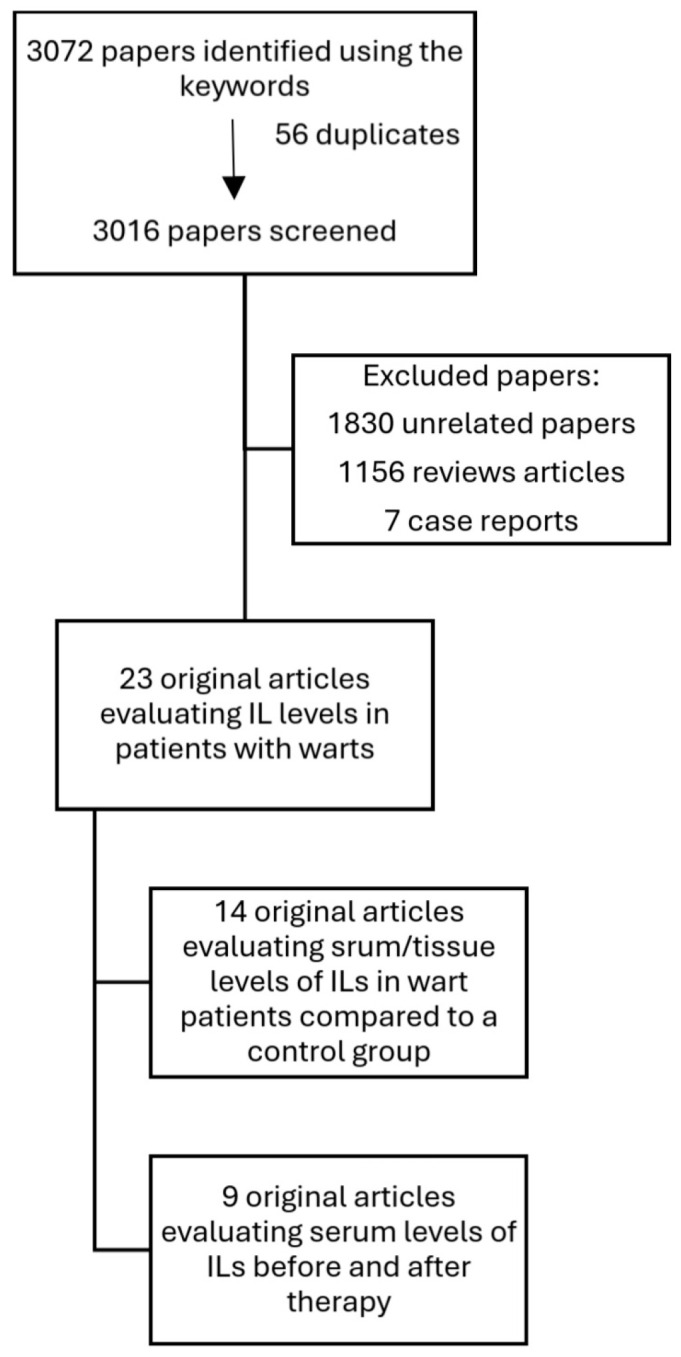
The selection criteria of the analyzed articles.

We conducted a narrative review consulting the PubMed and Google Scholar databases. We used “interleukin”, “wart”, “condyloma”, “HPV”, and “level” as keywords. We included original articles published between January 2015 and December 2024. We excluded reviews, clinical cases, and abstracts (Figure 3). We analyzed all studies that included patients with warts regardless of the clinical type of wart. A meta-analysis was considered unsuitable because of the significant variability among the studies.

## 3. Results

We identified 23 studies that we divided into two categories: 14 studies that evaluated serum/tissue levels of ILs in wart patients compared to a control group, and 9 studies that evaluated serum levels of ILs before and after therapy.

Table 1 shows the studies that analyzed interleukin levels in patients with warts compared to healthy individuals. Of the 14 studies reviewed, 6 focused on IL-17.

In all studies, measurements were performed on the subjects’ serum, except for one study in which tissue samples were analyzed.

**Table 1:** Interleukin levels in patients with warts compared to healthy individuals.

IL	Groups	Results	Conclusion	References
IL-17 IL-22	- Total of 50 patients with recalcitrant warts. - Total of 40 healthy controls.	Serum levels of IL-17 and IL-22 were *lower* in patients with warts compared to the control group (*p* < 0.001).	IL-17 and IL-22 could play a role in the pathogenesis of recalcitrant warts by altering the immune response and influencing the activity of immune cells.	Hussin et al. (2024) [25]
IL-36γ	- Total of 40 patients with warts (any clinical type). - Total of 40 healthy controls.	Serum levels of IL-36γ were *higher* in patients with warts compared to the control group (*p* < 0.001).	IL-36γ could modulate the cellular immune response in HPV-infected patients. IL-36γ could represent a therapeutic target, especially in immunosuppressed patients.	Al Sadik et al. (2023) [26]
IL-17	- Total of 40 patients with cutaneous warts and papillomas located on mucous membranes (oral, esophageal, and anal). - Total of 20 healthy controls.	Serum IL-17 levels were *higher* in patients with warts and papillomas compared to the control group (*p* = 0.004).	IL-17 can help HPV evade the host immune response and persist for a long time in keratinocytes.	Moustafa et al. (2023) [27]
IL-19	- Total of 50 patients with warts (common, plane, filiform, genital, periungual, and palmoplantar). - Total of 50 healthy controls.	Serum IL-19 levels were *lower* in patients with warts compared to the control group (*p* < 0.003).	IL-19 may play a critical role in the immune response against HPV.	Marie et al. (2023) [28]
IL-4	- Total of 40 patients with cutaneous warts. - 40 healthy controls.	Serum IL-4 levels were *higher* in patients with warts compared to the control group (*p* < 0.003).	IL-4 could play a role in the pathogenesis of warts.	Othafa et al. (2023) [29]
IL-17	- Total of 50 patients with warts (common, plane, filiform, genital, subungual, and palmoplantar). - Total of 50 healthy controls.	Serum IL-17 levels were *lower* in patients with warts compared to the control group (*p* < 0.001).	Low serum IL-17 levels alter the immune response mediated by Th1 cells, which increases susceptibility to HPV infection.	Nw et al. (2022) [30]
IL-33	- Total of 40 patients with genital warts. - Total of 40 healthy controls.	Serum IL-33 levels were *lower* in patients with genital warts compared to the control group (*p* < 0.01).	Low levels of IL-33 indicate a deficient immune response, which allows genital warts to develop and favors recurrences.	Eyada et al. (2022) [31]
IL-22	- Total of 20 patients with cutaneous warts. - Total of 20 healthy controls.	Serum IL-22 levels were *higher* in patients with warts compared to the control group (*p* < 0.001). IL-22 levels were significantly *higher* in patients with recurrent episodes compared to those who had warts for the first time (*p* = 0.007).	Increased production of IL-22 represents an adaptive mechanism against HPV infection.	Marie et al. (2021) [32]
IL-17	- Total of 60 patients with warts (common, palmar, plantar, and genital). - Total of 30 healthy controls.	Serum IL-17 levels were *higher* in patients with warts compared to the control group (*p* < 0.003).	IL-17 may contribute to the pathogenic mechanisms involved in the appearance of warts.	Alkady et al. (2020) [33]
IL-33	- Total of 25 patients with warts. - Total of 25 healthy controls.	IL-33 levels in lesional tissue were *higher* than in non-lesional tissue (*p* < 0.001). IL-33 tissue levels in patients with warts were *higher* compared to the levels measured in skin biopsies from the control group (*p* < 0.001).	Increased levels of IL-33 may represent a defense mechanism of the body against HPV infection. IL-33 could have a role as an adjuvant in HPV vaccines.	El-Rifaie et al. (2020) [34]
IL-17	- Total of 25 patients with recalcitrant warts. - Total of 25 healthy controls.	Serum IL-17 levels were *lower* in patients with warts compared to the control group (*p* < 0.003).	IL-17 contributes to the mechanisms involved in recalcitrant warts by altering the function of immune cells responsible for HPV clearance.	Ghanem (2020) [35]
IL-21 IL-33	- Total of 45 patients with genital warts. - Total of 45 healthy controls.	Serum levels of IL-21 and IL-33 were *lower* in patients with warts compared to the control group (*p* < 0.001).	Low serum levels of IL-21 and IL-33 can increase the risk of acquiring HPV infection and developing genital warts.	Abu El-Hamd et al. (2019) [36]
IL-6	- Total of 24 patients with palmoplantar warts. - Total of 28 healthy controls.	*No significant* differences in serum IL-6 levels were observed between the control group and patients with warts (*p* > 0.05).	Although serum IL-6 levels were not elevated, the presence of a pro-inflammatory response in these patients should not be excluded.	Mitran et al. (2019) [37]
IL-17	- Total of 60 patients with cutaneous warts. - Total of 20 healthy controls.	Serum IL-17 levels were *lower* in patients with warts compared to the control group (*p* < 0.001).	IL-17 is involved in the pathogenesis of HPV infection. Administration of IL-17 could have a role in therapy.	El-Hamd (2018) [38]

IL—interleukin, HPV—human papillomavirus.

**Figure 4:**
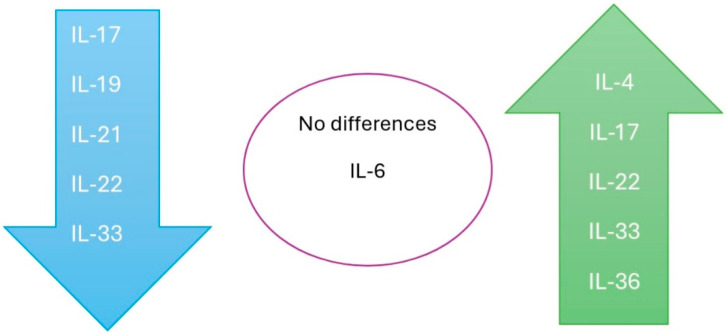
Serum variations in ILs in patients with warts compared to the control group.

Figure 4 presents a summary of the serum variations in the ILs in patients with warts compared to the control group. Regarding IL-17, some researchers have identified higher serum levels in patients with warts (two studies), while others have reported lower serum levels (four studies) among these patients compared to healthy individuals. Contradictory results were also observed for IL-22. In one study, serum levels were significantly lower in patients with warts compared to the control group, whereas another study reported higher levels. We identified only one study using tissue for IL level assessment (IL-33). In the serum-based studies, lower levels of IL-33 were identified in patients with warts compared to the control group; however, in tissue samples, the levels of IL-33 were higher compared to normal skin samples from healthy individuals.

Table 2 summarizes data on serum interleukin levels before and after treatment in patients with warts. The following treatments were administered: intralesional purified protein derivative—PPD (five studies); intralesional vitamin D3 (one study); intralesional *Candida* antigen (one study); Measles, Mumps, and Rubella—MMR vaccine (two studies); *Mycobacterium* w vaccine (one study); HPV bivalent vaccine (one study); high-frequency fulguration in association with the intramuscular administration of Bacillus Calmette–Guerin polysaccharide nucleic acid—BCG-PSN (one study); high-frequency fulguration in association with 5% imiquimod cream (one study); cryosurgery (one study); and electrosurgery (one study).

**Figure 5:**
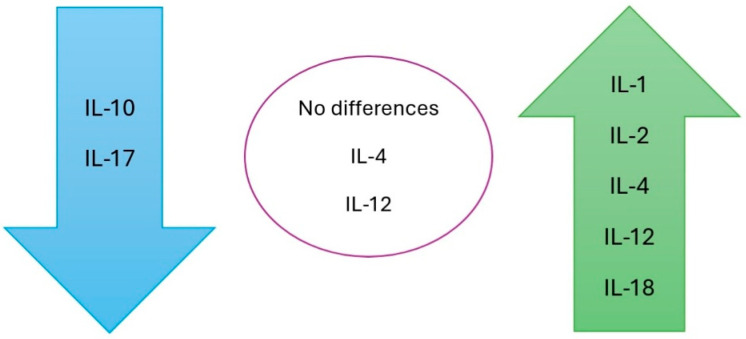
Serum variations in the ILs in patients with warts after therapy.

Figure 5 presents a summary of the serum variations in the ILs in patients with warts after therapy.

## 4. Discussion

Cytokines work synergistically and exhibit multiple overlapping and diverse functions [48,49]. Many cytokines undergo significant alterations in cervical precancerous lesions and neoplasia. Persistent HPV infection is associated with a pro-inflammatory status that fails to eliminate the virus and instead facilitates the progression of the disease. Elevated levels of certain cytokines, such as IL-6, IL-17, and IL-8, have been linked to tumor progression, whereas others, including IL-1, TNF-α, TGF-β, and IFN-α, play a role in suppressing HPV replication and tumor growth, especially during the early stages [50,51]. ILs constitute a large group of cytokines that play critical roles in immune modulation and inflammation [52]. The vast majority of the studies have focused on the roles of ILs in the pathogenesis of cervical cancer; however, in the context of warts, the studies are limited. Our review indicates that in the last 10 years, the following ILs were studied in patients with warts: IL-1, IL-2, IL-4, IL-6, IL-10, IL-12, IL-17, IL-18, IL-19, IL-21, IL-22, IL-33, and IL-36. Researchers have evaluated IL levels in patients with warts compared to a control group and have also assessed changes in these levels before and after therapy. Most studies that have analyzed IL before and after therapy evaluated immunotherapy as a therapeutic modality. There are no clear indications in the medical literature regarding immunotherapy in patients with warts, but these therapies are especially recommended in the case of recurrent, recalcitrant warts and in patients with a large number of warts or with warts located in areas that are difficult to treat such as the periungual or palmoplantar zones. Immunotherapy has the role of enhancing the recognition of the virus by the host’s immune cells, especially by activating the cellular immune response [53,54].

*IL-1.* Serum IL-1 levels were assessed in a single study conducted before and after immunotherapy. The levels of IL-1 significantly increased after intralesional PPD. It is well known that IL-1 activates the immune response, exerts a proinflammatory effect, and downregulates HPV gene expression, acting as an antiviral molecule [42]. IL-1 belongs to the IL-1 family and is found in two main forms: IL-1 alpha, which is normally expressed by epithelial and mesenchymal cells, and IL-1 beta, the expression of which is increased in pathological processes [55]. However, it has been shown that HPV-positive cells secrete less IL-1 beta than HPV-negative cells; the mechanisms are unclear, but E6 and E7 oncoproteins seem to be involved [56].

*IL-2*. We identified only one study that reported elevated IL-2 serum levels after therapy. IL-2 plays an important role in cellular immunity by supporting Th1 responses and exerting antitumor activity. However, the role of IL-2 in HPV infection remains incompletely elucidated. Recent studies have highlighted that IL-2 stimulates the proliferation of neoplastic cells in cervical cancer [57,58,59].

*IL-4*. We identified three studies that measured IL-4 levels in patients with warts. In one of the studies, the authors identified higher levels compared to controls, and another study highlighted that IL-4 levels increased after PPD immunotherapy. In the third study, it was shown that the bivalent HPV vaccine did not influence IL-4 levels in patients with warts. Singh et al. suggested that in patients with anogenital warts, the immune response shifts from a Th1 to a Th2 profile, which allows lesion progression. The examination of blood samples from these patients showed low levels of IFN-gamma-producing CD4+ and CD8+ T cells and high levels of IL-4-producing CD4+ T cells [60]. A recent study revealed that IL-4 mRNA levels are higher in patients with warts compared to healthy individuals [61]. The role of IL-4 in the pathogenesis of warts is further supported by a case report in which a patient with atopic dermatitis and warts treated with dupilumab, an IL-4 inhibitor, experienced the resolution of both atopic dermatitis lesions and warts shortly after treatment [62].

*IL-6.* Several studies have previously highlighted elevated serum levels of the pro-inflammatory cytokine IL-6 in patients with HPV infection [63,64]. A single study assessed IL-6 levels in patients with warts; the study, conducted by Mitran et al., did not reveal significant differences between patients with genital warts and healthy individuals [37]. In line with this, a study that included patients with cervical infection reported significantly higher IL-6 levels in HPV-negative patients compared to HPV-positive patients; however, the authors noted that the study included patients with recent infections, corresponding to early-stage cervical lesions [23].

*IL-10.* IL-10 exhibits anti-inflammatory properties, contributing to immune evasion and enabling an immunosuppressive microenvironment [65,66]. There are two studies available in the literature reporting lower levels of IL-10 in patients with warts after immunotherapy. Keratinocytes, dendritic cells, and regulatory T cells are the main immune cells that release IL-10. This IL can exert multiple effects on immune cells, such as suppressing the synthesis of proinflammatory cytokines, altering the differentiation of dendritic cells, and limiting the polarization of Th1 cells. HPV E6 and E7 proteins have been shown to stimulate IL-10 gene expression, leading to increased IL-10 levels, which in turn induce the expression of E6 and E7 proteins, thereby establishing a self-perpetuating cycle that supports viral replication and lesion progression [67].

*IL-12*. IL-12 was the most extensively studied interleukin when the response to various therapies was evaluated, especially immunotherapy; we identified four studies. Following cryotherapy and electrotherapy, no statistically significant differences were identified; however, in cases in which immunotherapy with PPD or MMR was employed, IL-12 levels were significantly increased post-therapy compared to pre-therapy levels. These differences may arise from the fact that electrosurgery and cryosurgery are destructive methods that do not exert effects on the immune system. On the other hand, immunotherapy directly affects the cells of the immune system.

The key cytokines that mediate Th1 responses are IL-12 and IFN gamma. IL-12 is released by macrophages and dendritic cells, exerting antiviral activity. In addition, IL-12 acts as an inducer of IFN gamma biosynthesis. Langerhans cells release high levels of IL-12 that act on naive T cells, inducing their activation and differentiation into Th1 cells, which in turn will release IFN gamma [41]. Together, IL-12 and IFN gamma are involved in the activation of cytotoxic T cells and NK cells, contributing to HPV clearance [43,44,46]. A recent study has highlighted that low serum levels of IL-12 represent a risk factor for infection with high-risk HPV types [68]. A recent study evaluated the potential genes that are involved in the modulation of the immune response in patients with genital warts. Thus, 56 samples were analyzed by immunohistochemical tests using antibodies against CD1a, FOXP3, CD3, CD4, CD8, and IFN. The increased expression of GZMB, IFNG, IL-12B, and IL-8 and the decreased expression of NFATC4 and IL-7 in genital wart samples were highlighted. The authors concluded that IL-8 and IL-12 exhibit a pro-inflammatory action and induce a Th1 response that is critical for viral clearance. NFAT plays a role in modulating the expression of cytokine genes predominantly in Th2 cells [69].

*IL-17*. IL-17 is the most studied interleukin in patients with warts. We identified seven studies with contradictory results. Some researchers have identified higher serum levels in patients with warts (two studies), while others have reported lower serum levels (four studies) among these patients compared to healthy individuals. One study highlighted the decrease in serum levels of IL-17 following the administration of the *Candida* antigen [39]. There are two theories that explain these contradictory results. The lower serum levels in patients with warts compared to controls could be due to the longer duration of the disease, which leads to IL-17 consumption [33]. On the other hand, the higher levels of IL-17 could result from a prolonged HPV infection that promotes an inflammatory process, leading to the release of elevated amounts of IL-17 [27]. In the studies included in our review, the disease duration was prolonged (several months).

A positive correlation was identified between serum levels of IL-17 and the duration of warts in a study conducted by Moustafa et al. [27]. In contrast, Nw et al. reported a negative correlation between serum IL-17 levels and both the number of warts and the duration of the disease [30]. Alkady et al. did not find a correlation between the number of warts, the duration of the disease and serum levels of IL-17 [70]. IL-17 stimulates the function of cytotoxic T lymphocytes, which contributes to HPV clearance. Thus, low serum levels of IL-17 represent a condition that facilitates HPV infection and the subsequent appearance of warts [30,35]. In the case of patients with recalcitrant warts, HPV infection is persistent, implying a long-term struggle between immune system cells and HPV, resulting in decreased IL-17 levels. Under such conditions, the immune responses mediated by Th1 cells are altered, shifting toward a Th2-mediated immune response, which allows the virus to multiply and persist in tissues [25].

HPV has the ability to activate the STAT3 pathway, which stimulates the release of IL-17, resulting in keratinocyte proliferation and increased viral replication [27]. It is known that IL-17 is a cytokine with a pro-inflammatory effect and plays an important role in viral clearance. However, in situations in which the level of IL-17 is increased, the so-called hyper-inflammation phenomenon occurs, which underlies the development of numerous inflammatory diseases and cancer. IL-17 remains a cytokine surrounded by several controversies. Some studies have shown that it plays an important role in eliminating malignant cells, while others suggest that it can contribute to their dissemination. In the case of cutaneous warts, satisfactory results have been observed following therapy with agents that block its activity, but exacerbations can also occur [71].

*IL-18*. Studies have shown a synergistic effect between IL-18 and IL-12, enhancing IFN-gamma release and the cytotoxic action of NK and cytotoxic cells. In this review, we have identified only one study regarding IL-18. Notably, IL-18 levels were significantly higher in patients with warts who responded to treatment compared to those who did not respond, which denotes the role of IL-18 in the lesion clearance [40].

*IL-19*. We have identified only one study that analyzed IL-19 in patients with warts. Marie et al. reported lower levels in patients with warts regardless of the number of lesions compared to healthy controls [28]. However, there were higher serum IL-19 levels in patients with a larger number of warts compared to patients with fewer warts or a single lesion. It is worth noting that almost half of the patients in this study had recurrent warts [28]. These results may suggest that in patients with higher levels of IL-19, the immune response was switched to Th2, which allowed the virus to replicate [28]. Interestingly, IL-17 has been observed to stimulate IL-19 synthesis in keratinocytes [72]. It has been shown that high-risk HPV types can alter the immune response by shifting it from a Th1 profile to a Th2 profile, thereby promoting disease progression [21].

*IL-21*. IL-21 is a multifaceted molecule that has multiple roles in the regulation of the immune response, including the modulation of cytotoxic T cells and NK cells and the promotion of Th1 cell differentiation [73]. However, some authors suggest that IL-21 is a cytokine associated with Th2 cells, thereby suppressing the differentiation of naive Th cells into Th1 cells that release IFN-gamma [74]. IL-21 contributes to the antiviral response, especially in chronic infections, by enhancing B cell function and influencing antibody production [75,76]. Only one study evaluating IL-21 levels in patients with warts is available in the recent literature [36]. The same study showed no correlation between IL-21 levels and the number of warts.

*IL-22*. The results regarding serum IL-22 levels are contradictory. In one study, serum levels were significantly lower in patients with warts compared to the control group, whereas another study reported higher levels. Both studies identified a positive correlation between IL-22 levels and the number of warts. There are some differences between the two studies. Hussin et al. included only patients with recalcitrant warts [25], whereas Marie et al. analyzed a heterogeneous group of patients in terms of treatment response [32]. Another difference is the duration of the disease, which was significantly longer in a study carried out by Marie et al. It was observed that in patients with chronic lesions, the levels of IL-22 are higher, which could be one of the reasons why Marie et al. obtained higher levels compared to Hussin et al. The protective or pathogenic role of IL-22 is dependent on factors such as the type of virus involved in the infectious process, the severity of the disease, and the expression of IL-17A. IL-22, a member of the IL-10 family produced mainly by Th1, Th17, and Th22 cells, exhibits a dual effect, “friend or foe”, in various viral infections. Immune cells lack receptors for IL-22 [32,77]. Since keratinocytes express receptors for this interleukin, IL-22 is involved in the pathogenesis of various skin diseases such as atopic dermatitis or psoriasis, but its role in cutaneous warts has been poorly studied [32,78].

*IL-33*. IL-33, a member of the IL-1 family, modulates the Th2 immune response and serves as an important immunomodulator in allergic, infectious, or autoimmune diseases [79]. We have identified three studies on IL-33. In two studies, the serum levels were measured, and in one, the levels in the lesional tissue were analyzed. In serum-based studies, lower levels were identified in patients with warts compared to the control group; however, in tissue samples, the levels of IL-33 were higher compared to normal skin samples from healthy individuals. These results are in concordance with the results obtained by Jin et al., who analyzed samples from three patients with herpes virus infection and from two patients with vulgar warts. In the case of patients with warts, IL-33 expression was very low compared to those with herpes infection, in which an increased expression was observed. The authors suggested that this difference may be based on the immune response that the virus generates in the epidermis [80]. Moreover, it is known that IL-33 is produced as a defense mechanism against tissue damage (inflammation, infection, etc.) [81].

No correlations were observed between serum IL-33 levels and the number of genital warts or between serum IL-33 levels and recurrence episodes [31,38]. Similarly, El-Rifaie et al. did not identify a correlation between the duration of lesions and IL-33 tissue levels [34].

*IL-36*. The IL-36 cytokine group, a member of the IL-1 superfamily, comprises IL-36α, IL-36β, and IL-36γ. IL-36α and IL-36γ are expressed by various cells in the skin, including keratinocytes, macrophages, and fibroblasts, among others, and are involved in host defense [82]. In this review, we identified only one study that evaluated serum IL-36γ levels. The authors found no correlations between IL-36γ levels and patient characteristics such as sex and age, or with the number, location, clinical appearance, or recurrence rate of warts [26]. A metaproteomic study involving the analysis of cervical biopsies and cytology showed that the proteins lumican and galectin-1 have the highest expression levels in biopsy samples, whereas IL-36 and IL-1RA showed the highest expression levels among human proteins in the cytology samples [83].

Further studies involving a larger number of patients with similar characteristics are needed to determine whether the serum levels of ILs may be used as markers of disease severity. The analyzed studies show that for certain ILs, such as IL-21, IL-33, and IL-36, no correlations were found with the number of lesions, whereas for others, such as IL-17, IL-19, and IL-22, statistically significant correlations with the number of lesions were observed, which is in line with the fact that the immune response has an essential role in the progression of HPV infections [22].

Regarding the potential role of ILs in therapy, data are scarce. El-Rifaie et al. suggest that IL-33 could be used as an adjuvant in the HPV vaccine [34]. Villarreal et al. were the first to demonstrate that IL-33 could serve as a molecular adjuvant in a vaccine to stimulate protective immunity. They refined a DNA vaccine encoding mtrIL-33 and tested its efficacy when used as an adjuvant together with a DNA construct encoding the HPV16 antigens E6 and E7 [84].

El-Hamd et al. suggest that both the topical and systemic administration of IL-17 could be useful in the therapy of skin warts. Models with IL-17 deficiency are more susceptible to bacterial and viral infections [38]. Sun et al. reported the case of a patient with psoriasis treated with secukinumab (an IL-17 inhibitor) who experienced a rapid progression of genital warts under treatment [85]. However, recently, in a group of psoriasis patients undergoing therapy with secukinumab, a regression of warts was observed [86]. These results are consistent with the contradictory results presented in Table 1. Of the 25 studies reviewed in this article, 16 were published in the last 5 years, indicating a growing interest in this topic. It should be emphasized that in most studies, the number of patients included was small and only one type of interleukin was analyzed. Studies that include a higher number of patients and a larger panel of interleukins to be evaluated in the same group of patients are needed. Regarding therapies, very diverse therapies were analyzed, which led to heterogeneity of results.

## 5. Conclusions

The pathogenesis of HPV infection is complex and further investigation into the role of ILs could offer new insights. Currently, there are few studies, and the data are unclear and, in some cases, contradictory. Data from the analyzed studies can be used to identify new therapies and markers that correlate with disease severity. However, multicenter studies with a larger number of patients and clear, non-standardized methodologies are needed. The role of ILs in the pathogenesis of HPV is supported by the results of studies that evaluated their levels before and after therapy. These studies found that the levels of IL-1, IL-2, IL-4, IL-12, and IL-18 increased, while the levels of IL-10 and IL-17 decreased after therapy. Additional research is needed to clarify all aspects of the involvement of ILs in the pathogenesis, persistence, and resolution of warts.

## Figures and Tables

**Table 2:** Serum interleukin levels before and after treatment in patients with warts.

IL	Groups	Results	Conclusion	References
IL-17	- In total, 60 patients with common warts were treated with *Candida* antigen, intralesional administration. - In total, 30 patients were treated with saline solution.	A significant *decrease* in serum IL-17 levels was observed after treatment with *Candida* antigen (*p* < 0.01).	IL-17 levels are modulated by the *Candida* antigen. Thus, serum IL-17 levels can be considered a marker for assessing response to treatment.	Nassar et al. (2022) [39]
IL-18	- In total, 25 patients with warts (all clinical types) were treated with PPD, intralesional administration. - In total, there were 25 healthy controls.	A significant *increase* in serum IL-18 levels was observed after treatment (*p* = 0.025). Regarding the comparison with the control group, there were no statistically significant differences in the pre-treatment stage, but statistically significant differences were observed in the post-treatment stage (*p* = 0.036).	IL-18 modulates the immune response induced by PPD and promotes the remission of warts.	Korsa et al. (2022) [40]
IL-4	- In total, 40 patients with recalcitrant warts (cutaneous and anogenital) received bivalent HPV vaccine. - In total, there were 40 healthy controls.	Blood samples were cultured with and without the bivalent HPV vaccine. In cultures stimulated with the bivalent HPV vaccine, IL-4 levels showed a slight decrease that was *not statistically significant* (*p* > 0.05).	The HPV vaccine does not induce an IL-4 mediated response in patients with warts.	Hammad et al. (2021) [41]
IL-1 IL-10	- In total, 36 patients with cutaneous warts (12 patients received PPD, 12 patients MMR vaccine, and 12 patients - *Mycobacterium* w vaccine).	An *increase* in serum IL-1 levels was observed after immunotherapy in all groups, differences being statistically significant only in the group that received PPD (*p* = 0.008). A *decrease* in serum IL-10 levels was observed after immunotherapy in all groups, differences being statistically significant in the group that received PPD (*p* = 0.027) or MMR (*p* < 0.01).	Both Th1 and Th2 cytokines are influenced by immunotherapy.	Sil et al. (2021) [42]
IL-12	- In total, 18 patients with multiple extragenital warts treated with cryosurgery. - In total, 13 patients with multiple extragenital warts treated with electrosurgery.	There were *no differences* between serum IL-12 levels in the 2 groups pre- and post-therapy (*p* > 0.05).	IL-12 plays a role in HPV clearance; however, studies on a larger number of patients are needed.	Awad et al. (2020) [43]
IL-12	- In total, 23 patients with extragenital warts treated with intralesional PPD. - In total, 22 patients with extragenital warts treated with intralesional vitamin D3.	*Higher* serum IL-12 levels were identified in patients after the treatment with intralesional PPD (*p* = 0.034). Regarding vitamin D3, no statistically significant differences were observed (*p* = 0.368).	IL-12 contributes to the clearance of HPV, but further research involving a larger patient population is necessary to confirm this finding.	Abou-Taleb et al. (2019) [44]
IL-2 IL-10	- In total, 56 patients with genital warts treated with high-frequency fulguration in association with intramuscular administration of BCG-PSN. - In total, 56 patients with genital warts treated with high-frequency fulguration in association with 5% imiquimod cream.	Serum IL-2 levels were *higher* and IL-10 levels were *lower* after therapy in both groups (*p* < 0.001).	Elevated levels of IL-2 and decreased levels of IL-10 may contribute to the regression of warts.	Chen et al. (2017) [45]
IL-12	- In total, 25 patients with multiple warts treated with intralesional PPD.	There were *no differences* between serum IL-12 when compared before and after therapy (*p* > 0.05).	The study of interleukins in tissue is necessary to better evaluate their role in warts.	El-Samahy et al. (2016) [46]
IL-4 IL-12	- In total, 30 patients with cutaneous and genital warts (10 patients received PPD, 10 patients received MMR vaccine, and 10 patients received saline solution).	After treatment, *higher* serum levels of IL-4 and IL-12 were recorded among patients treated with PPD and MMR compared to those treated with saline solution (control group) (*p* < 0.05).	IL-4 and IL-12 are involved in the immune response induced by PPD and MMR, but considering that a complete resolution of the lesions did not occur, other molecules are probably involved.	Shaheen et al. (2015) [47]

PPD—purified protein derivative; MMR vaccine—Measles, Mumps, and Rubella vaccine; BCG-PSN—Bacillus Calmette–Guerin polysaccharide nucleic acid; HPV—human papillomavirus.

## Data Availability

Not applicable.
